# MOP Reduction During Long-Term Methamphetamine Withdrawal was Restored by Chronic Post-Treatment with Fluoxetine

**DOI:** 10.2174/157015911795017056

**Published:** 2011-03

**Authors:** H Yamamoto, Y Takamatsu, K Imai, E Kamegaya, Y Hagino, M Watanabe, T Yamamoto, I Sora, H Koga, K Ikeda

**Affiliations:** a Division of Psychobiology, Tokyo Institute of Psychiatry, Tokyo, Japan; b Laboratory for Medical Genomics, Department of Human Genome Technology, Kazusa DNA Research Institute, Chiba, Japan; c Department of Anatomy, Hokkaido University School of Medicine, Sapporo, Japan; d Molecular Recognition, Yokohama City University, Yokohama, Japan; e Department of Psychobiology, Tohoku University, School of Medicine, Sendai, Japan

**Keywords:** Methamphetamine, conditioned place preference, gene expression, withdrawal, fluoxetine, mu-opioid receptor, frontal cortex, mice.

## Abstract

Previously, we found fluoxetine reduces methamphetamine preference in mice. However, effects of fluoxetine on developed methamphetamine preference and on methamphetamine induced gene expression changes have been largely unknown. The present study investigates effects of post-treatment with fluoxetine on methamphetamine dependence and on gene expressions after long-term withdrawal in mice. First, we examined whether chronic post-treatment with fluoxetine attenuated methamphetamine-conditioned place preference. Next, we examined the changes in gene expression levels after long-term withdrawal (with saline or fluoxetine treatment) following chronic methamphetamine treatment. Using mRNA from the pooled frontal cortices of 10 mice per group, gene expression analyses were performed using a custom-developed cDNA array and a real-time quantitative reverse transcription-PCR. Chronic post-treatments with fluoxetine abolished the conditioned place preference developed by methamphetamine administrations. Even after long-term withdrawal from repeated methamphetamine administration, µ-opioid receptor (MOP) gene expression was significantly reduced in the frontal cortex. The reduced MOP gene expression in the frontal cortex was restored by chronic administration with fluoxetine. These changes were confirmed by Western blot analyses. These findings suggest that the chronic post-treatments with fluoxetine might be effective for restoring the reduction of MOP levels in the frontal cortex following long-term abstinence from methamphetamine.

## INTRODUCTION

The development process of sensitization to the behavioral effects of psychostimulants is well-researched. There is substantial evidence that the mesocorticolimbic dopamine system and its excitatory glutamatergic inputs are critical [1, 2]. However, glutamate antagonists do not block the expression of sensitization [3]. Similarly, dopamine antagonists can block the development of sensitization to psychostimulants without blocking its expression [4]. On the other hand, glutamatergic afferents from the prefrontal cortex to the ventral tegmental area and the nucleus accumbens have been reportedly implicated in both the development and expression of sensitization to cocaine and amphetamine [5]. The frontal cortex is important region that is activated in addicted subjects during intoxication, craving, and bingeing, and deactivated during withdrawal [6].

Currently, effective pharmacotherapy for psychostimulant abuse has not been established. However, preclinical studies have indicated that the serotonergic system can effectively modulate the behavioral effects of amphetamine. That is, a negative relationship was observed between the potencies of several cocaine- and amphetamine-like drugs in self-administration studies and their binding affinities for serotonin uptake sites [7, 8]. Administration of the serotonin uptake inhibitor fluoxetine decreased self administration of amphetamine [9] in rodents. Amphetamine withdrawal elevates brain reward threshold in rats [10]. Harrison *et al.* (2001) [11] have reported that co-administration of a 5-HT_1A_ receptor antagonist and fluoxetine reverses reward deficits observed during nicotine or amphetamine withdrawal. These findings suggest that increasing brain serotonin activity can attenuate the behavioral and reinforcing effects of amphetamines.

In the present study, we used the frontal cortices of chronically methamphetamine-injected mice to explore molecules that expressions were changed during long-term abstinence and fluoxetine reversed its expressional changes. First, we applied comprehensive approach to exploration of candidate genes by using cDNA array system utilizing mouse KIAA-homologous cDNA (mKIAA) clones. Next, gene expressions and protein expressions were examined by real-time quantitative reverse transcription-polymerase chain reaction (qRT-PCR) experiments and immunoblot analyses, respectively.

## MATERIALS AND METHODS

### Animals

Ten-week-old male C57BL/6J mice were purchased from CLEA Japan (Tokyo, Japan). The experimental procedures and housing conditions were approved by the Tokyo Institute of Psychiatry Institutional Animal Care and Use Committee, and all animals were cared for and treated humanely in accordance with our institutional guidelines on animal experimentation.

### Conditioned Place Preference Test

The conditioned place preference test was performed according to the method of Hoffman and Beninger (1988) [12] with some modifications. For this test, we used a two-compartment Plexiglas chamber (Neuroscience Inc., Osaka, Japan). We selected a counterbalanced protocol to nullify each mouse’s initial compartment preference [13].

Acquisition of methamphetamine-induce place preference was shown in drug-naive mice. On Day 1, the mice (*n* = 18 - 20 per group) were allowed to freely explore the two compartments for 15 min. On Day 2, the same trial was performed, and the time spent in each compartment and shuttle numbers were measured for 15 min. Conditioning was conducted once daily for four consecutive days (Days 5-8). Mice were intraperitoneally (i.p.) injected with methamphetamine (2 mg/kg) and immediately confined to the black or white compartment for 50 min on Day 5. On Day 6, the mice were injected with saline and immediately confined to the opposite compartment for 50 min. On Days 7 and 8, the same conditioning as on Days 5 and 6 was repeated. After methamphetamine conditioning, the mice received saline or fluoxetine (20 mg/kg, i.p.) once a day for 10 days (Days 9–18). On the last day (Day 19), the mice were not treated with saline or fluoxetine. The time spent in each compartment and shuttle numbers were measured for 15 min without methamphetamine injection. Time spent in the drug-paired compartment during pre- and post-conditioning preference tests were analyzed by within-group paired *t*-tests.

### Tissue Preparation, RNA Isolation, Probe Labeling, and Microarray Hybridization

For analysis of gene expression studies, mice in the long-term withdrawal groups were given a saline or methamphetamine injection (2 mg/kg, i.p.) once a day for 14 days, housed for 7 days without any injection, and then injected with saline or fluoxetine (20 mg/kg, i.p.) once a day for 14 days and sacrificed 24 h after the last injection.

After decapitation, the frontal cortices from 10 mice per treatment group were quickly dissected on ice, immediately frozen at -80°C, and used as a pooled sample for the cDNA array experiment [14], qRT-PCR analysis and used as separate samples for western blot analysis.

### qRT-PCR

To confirm the cDNA array results, qRT-PCR was performed on the MOP gene using the TaqMan strategy (Mm01188089 m1) and the ABI Prism 7300 Sequence Detection System (Applied Biosystems, Foster City, CA). For the expression of the genes other than the MOP gene, real-time qRT-PCR was performed using a cybergreen fluorescence-based assay kit (SBYR Green RT-PCR kit; Takara Bio Inc., Shiga, Japan) according to the manufacturer’s instructions. The levels of all cDNAs generated from mRNA by reverse transcription were calculated by the standard curve method for quantification and normalized with respect to GAPDH transcript levels.

### Western Blotting

P_2_ membranes were prepared from homogenate derived from each frontal cortex. Samples were mixed with an equal volume of Laemmli's samples buffer (10 µg/lane), boiled for 3 min and then resolved on a 5-20% gradient SDS polyacrylamide gel electrophoresis. The proteins were electrotransferred onto PVDF membranes in a semi-dry blotter.

We used two rabbit polyclonal antibodies specific for MOP. *N*-terminal-specific antiserum (N-38) was prepared against 1–38 amino acids sequences of the MOP *N*-terminus [15]. The *C*-terminus-specific antibody (AB5511, lot No. 25050663) was purchased from Chemicon International (Temecula, CA, USA). Rabbit polyclonal anti-actin antibody was purchased from Sigma-Aldrich (St. Louis, MO, USA) and used to detect endogenous actin as an internal standard.

### Statistical Analysis

Parametric analysis of quantitative data was performed using a one-way analysis of variance (ANOVA) followed by Scheffe's test. Nonparametric analysis was conducted using the Kruskal-Wallis test followed by Scheffe's *post hoc* comparison test. The level of statistical significance was set at *p* < 0.05.

## RESULTS

### Effects of Chronic Administration of Fluoxetine on Methamphetamine-Induced Conditioned Place Preference

Time spent in the conditioned compartment was significantly increased when saline was administered for 9 days after methamphetamine conditioning (*n* = 20, *t* = 4.408, *p* = 0.0003; Fig. (**1A**)). By contrast, time spent in the conditioned compartment was not significantly changed when fluoxetine (20 mg/kg) was administered for 9 days after methamphetamine conditioning (*n* = 18, *t* = 1.513, *p* = 0.1488; Fig. (**1B**)). These results suggest that subchronic administration of fluoxetine at a dose of 20 mg/kg to mice weakened the place preference induced by methamphetamine. Thus next, we used mice chronically treated with fluoxetine (20 mg/kg) during methamphetamine withdrawal in the gene expression and western blot analyses.

### Effects of Fluoxetine on Methamphetamine-Induced Changes in Gene Expression after Long-Term Withdrawal

In the cDNA array experiments, expressions of a few genes, MOP, N-methyl-D-aspartate (NMDA) receptor 2D subunit (NR2D), nociceptin receptor, G protein-activated inwardly rectifying K^+^ channels (GIRKs) and inward rectifier K^+^ channel Kir2.3 (IRK3), were reduced after 3 weeks withdrawal following chronic methamphetamine treatment (MAP-Saline column in Table **1**). These reductions (< 70 % reduction in the saline treatment) were recovered in some genes except GIRK1 and GIRK3 when treated with fluoxetine for 2 weeks after the chronic methamphetamine treatment (MAP-Flx in Table **1**). The cDNA array experiments were performed in multiple determinations using a set of pooled samples.

To assess data derived from cDNA array, we performed qRT-PCR analyses on these gene expressions using mRNA derived from three sets of pooled cortices. The qRT-PCR data were shown in parenthesis in Table **1**. Expressions of mu-opioid receptor and IRK3 were approximately similar between cDNA array and qRT-PCR analyses. However, gene expressions of NR2D, nociceptin receptor and GIRK2 were not reduced after 3 weeks withdrawal following chronic methamphetamine treatment (parenthesis of MAP-Saline column in Table **1**). On the other hand, reduced expression (MAP-Saline) of mu-opioid receptor was recovered by fluoxetine treatment til 93%, while that of IRK3 was 35% (parenthesis of % recovery by fluoxetine treatment in Table **1**).

Results using cDNA array experiment and qRT-PCR analyses had shown that the reduced gene expression of MOP in the frontal cortex during long-term withdrawal was restored by subsequent fluoxetine treatments.

### Fluoxetine Effects on MOP-Immunoreactivity

To investigate the results obtained from the gene expression analyses, immunoblot of MOP in each frontal cortex of methamphetamine-injected mouse was performed (4 – 12 mice per group). MOP-immunoreactivity (IR) in the frontal cortex was detected as a broad band at a position consistent with a molecular weight of 65,000 with anti-MOP sera (N-38 and AB5511) (Fig. (**2A**)). These two anti-MOP sera (N-38 and AB5511) were specific against MOP molecule. The detected broad bands by these antisera were abolished using MOP knockout mice. MOP-IR with AB5511 antibody of the MAP-Saline sample (3-week withdrawal with saline injections after chronic methamphetamine injections, *n* = 11) was significantly lower than that of the Saline-Saline sample (3-week withdrawal with saline injections after chronic saline injections, *n* = 12) (*p* = 0.0101; Fig. (**2B**)). The intensity of MOP-IR in the MAP-Flx sample (3-week withdrawal with fluoxetine injections after chronic methamphetamine injections, *n* = 4) was significantly higher than that in the MAP-Saline sample (*p* = 0.0203; Fig. (**2B**)) and not different from that in the Saline-Saline sample (*p* = 0.902). The intensity of MOP-IR in the Saline-Flx sample (3-week withdrawal with fluoxetine injections after chronic saline injections, *n* = 6) was not significantly different from those in the Saline-Saline and MAP-Flx samples (*p* = 0.699; *p* = 0.473). These results have shown that MOP-IR was reduced after 3 weeks withdrawal following chronic methamphetamine treatment and this reduction was recovered by subsequent fluoxetine treatments.

## DISCUSSION

In the present study, repeated methamphetamine administration induced a conditioned place preference. The place preference was significantly attenuated by chronic fluoxetine treatments during long-term withdrawal. Fluoxetine is reported to reverse reward deficits during amphetamine withdrawal [11]. Recently, Kaneko *et al.* (2007) [16] reported that 5-day treatment with fluoxetine and paroxetine during methamphetamine withdrawal may at least in part reverse methamphetamine-induced behavioral sensitization. Taken together, these results suggest that chronic fluoxetine treatment can partially reverse methamphetamine-induced behavioral changes.

We observed that 3-week withdrawal after chronic methamphetamine induced gene expression changes. Of interest, both in the cDNA array and the real-time qRT-PCR analyses, MOP gene expression was decreased in the frontal cortex after long-term withdrawal, and partially restored by chronic fluoxetine treatment during methamphetamine withdrawal. On the basis of these results of gene expressions, we have performed the protein expressions of MOP by western blot analysis. Of interest, immunoreactive MOP level was significantly reduced during methamphetamine withdrawal, while chronic fluoxetine administrations during withdrawal could partially restore the reduced MOP expression level in the frontal cortex.

To date, changes in MOP expression after withdrawal from alcohol and amphetamine have been reported. Lower MOP binding potential in the right dorsal lateral prefrontal cortex, the right anterior frontal cortex, and the right parietal cortex is associated with higher craving in male alcohol-dependent subjects undergoing alcohol withdrawal [17], even though after long-term abstinence alcoholic patients display no changes in the prefrontal cortex but an increase in the binding potential of MOP in the ventral striatum, including the nucleus accumbens [18]. In contrast, subchronic injections of amphetamine resulted in a significant reduction in MOP mRNA levels in the nucleus accumbens shell [19], whereas no significant changes were observed in the level of MOP mRNA expressed in the nucleus accumbens shell of behaviorally sensitized rats tested 2 or 14 days after withdrawal [20]. Recently, Chiu *et al.* (2006) [21] have used the whole brain except cerebellum repeatedly injected with 2.5 mg/kg of methamphetamine for 7 days and reported that maximal binding of MOP is not changed on days 2 and 5, but down-regulated on day 8. After cessation of drug treatments, the maximal binding of MOP returns to normal level on day 11 and up-regulates on day 21. These data are of interest considering that the expressions of behavioral sensitization were attenuated by pretreatment with 10 or 20 mg/kg of naltrexone either during the induction period or before methamphetamine challenge when they were tested on days 11 and 21 [22]. However they also mention that whole brain samples may be insufficient to reveal the region-specific changes [21]. Investigating methamphetamine-induced craving, endogenous opioid system is involved in the mechanisms underlying cue-induced relapse [23]. Naltrexone inhibits reinstatement of drug-seeking behavior induced by methamphetamine-associated cues, but has no effect on methamphetamine-priming-induced reinstatement. The implication of these results is that there are distinct mechanisms underlying drug-seeking behavior induced by re-exposure to drug-associated cues and that induced by drug priming. Further, the results indicate that increasing activity of the opioid system is involved in the cue-induced drug-seeking behavior, but not in that induced by drug-priming [23]. Naltrexone pretreatment also attenuates context-induced alcohol seeking and inhibits c-fos mRNA expression in the basolateral amygdala and the CA3 subregion of the hippocampus [24]. Therefore, regional specificity is important to study methamphetamine induced behaviors, including conditioned place preference, drug-seeking behavior and behavioral sensitization. To our knowledge, this is the first report of reduced MOP expression in the frontal cortex of long-term methamphetamine withdrawal detected by gene expression and protein expression analyses. This result would provide important insight into the relationship between mu-opioid receptor in the frontal cortex and methamphetamine induced behaviors.

In humans, Ide *et al. *(2006) [25] have shown associations between MOP gene (*OPRM1*) polymorphisms and methamphetamine dependence/psychosis. They also found significant differences in both genotype and allele frequencies of single-nucleotide polymorphisms (SNPs) in the *OPRM1* gene between control and methamphetamine-dependent/psychotic patients. There also is a significant association between SNPs and patients with transient psychosis. These findings suggest that MOP function may affect the development of methamphetamine psychosis. MOP also may be a key molecule involved in the mechanisms underlying behavioral changes after long-term withdrawal following chronic methamphetamine treatment.

In conclusion, the present study showed that reduced mRNA and protein expressions of MOP gene were found in the frontal cortex of methamphetamine-abuse model mice even after long-term abstinence. Furthermore, the subchronic fluoxetine treatment during methamphetamine withdrawal restored MOP expressions. Although the mechanisms underlying the therapeutic effect of fluoxetine for methamphetamine abuse should be further investigated, we suggest a possibility that the restoration of MOP expression can be used as one of therapeutic markers for drug dependence.

## Figures and Tables

**Fig. (1):**
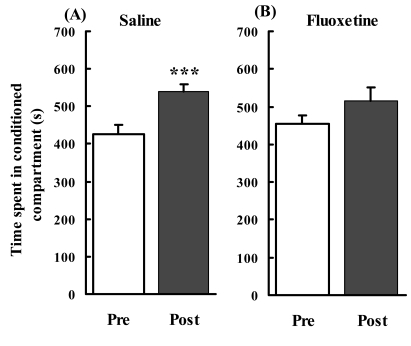
Effects of chronic administration of fluoxetine on the established methamphetamine-induced conditioned place preference. After methamphetamine conditioning, mice received (**A**) saline or (**B**) fluoxetine for 9 days. Each bar represents mean ± SEM of the time spent in the methamphetamine-paired compartment during a 15-min test session. Pre: preference test during the pre-conditioning phase. Post: preference test during the post-conditioning phase. ***: difference between Pre and Post, *p* < 0.001.

**Fig. (2):**
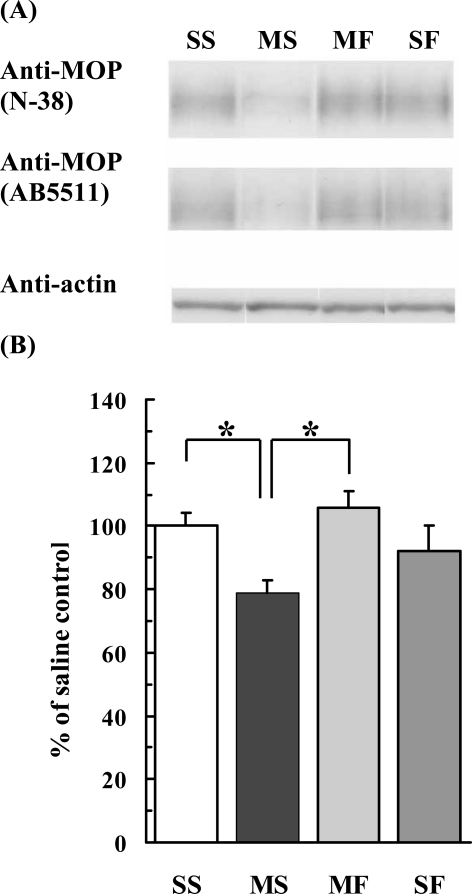
Immunoblot analyses of changes in MOP protein levels in the frontal cortex by saline or methamphetamine treatment followed by saline or fluoxetine treatment. (**A**) Typical immunoblot with anti-MOP and anti-actin antibodies. Using antiserum selective for MOP *N*-terminal (N-38) or *C*-terminal (AB5511), similar broad bands were detected using the same membrane. Actin levels were measured as references. (**B**) Quantitation of densitometer values using antiserum (AB5511). Values were normalized using actin values and represented as percentage of saline control values. Each bar represents the mean ± SEM of more than four individual mouse frontal cortices. Treatment for 2 weeks with saline followed by two weeks with saline (saline-saline [SS] sample, *n* = 12); treatment for 2 weeks with methamphetamine followed by 2 weeks with saline (methamphetamine-saline [MS] sample, *n* = 11); treatment for 2 weeks with methamphetamine followed by 2 weeks with fluoxetine (methamphetamine-fluoxetine [MF] sample, *n* = 4); and treatment for 2 weeks with saline followed by 2 weeks with fluoxetine (saline-fluoxetine [SF] sample, *n* = 6). *: difference between methamphetamine-saline group and saline-saline group or methamphetamine-fluoxetine group, *p* < 0.05, ANOVA.

**Table 1:** Effect of Chronic Fluoxetine Treatment on Changes in Gene Expression After Long-Term Withdrawal Following Chronic Methamphetamine Injections

cDNA Array Result (RT-PCR Result)						
Entrez		Gene(Property)	MAP-Saline	MAP-Flx	Saline-Flx	% of Recovery
Official Symbol	Gene ID			(% of Saline-Saline Control)		by Flx Treatment
** Receptors**						
Htr2c	15560	5HTR1C	88.5	78.3	88.9	60.0 (92.8)
Htr1e	107927	5HTR1E	94.8	85.5	107.6	
Htr2a	15558	5HTR2A	108.3	102.6	113.1	
Adra2a	11551	alpha2AR	115.5	93.8	97.5	
Bzrap1	207777	benzodiazapine receptor (peripheral) associated protein 1	88.0	90.0	82.4	
Oprd1	18386	delta-opioid receptor	74.2	90.0	90.0	
Esrrg	26381	estrogen-related receptor gamma	80.4	74.6	83.4	
Oprk1	18387	kappa-opioid receptor	80.4	101.8	115.1	
Lepr	16847	leptin R	95.8	90.7	99.3	
Sigmar1	18391	Sigma-1 receptor	87.9	83.4	96.5	
Oprm1	18390	mu-opioid receptor	58.5 (75.0)	83.4 (98.2)	86.4 (90.1)	
Grm1	14816	mGluR1	105.0	117.5	120.5	
Grm5	108071	mGluR5	84.3	82.6	98.7	
Npy2r	18167	neuropeptideY-Y2 receptor	93.4	93.2	84.5	
Grin2b	14812	NR2B	70.1	78.0	96.1	
Grin2c	14813	NR2C	85.9	79.7	92.3	
Grin2d	14814	NR2D	60.8 (95.3)	64.5 (80.7)	84.2 (88.2)	9.4
Grin1	14810	NR1	88.9	104.8	95.7	
Oprl1	18389	nociceptin receptor	68.8 (102)	91.1 (92.2)	81.5 (92.4)	71.6
Ogfr	72075	opioid growth factor receptor	94.2	105.9	112.7	
** Ion Channels**						
Cacna2d2	56808	calcium channel, voltage-dependent,	86.4	103.2	90.8	38.2
Kcnj3	16519	GIRK1	63.4	54.4	70.9	
Kcnj6	16522	GIRK2	66.1 (92.5)	79.0 (106)	78.7 (98.3)	
Kcnj9	16524	GIRK3	57.5	54.4	71.9	
Kcnj12	16515	IRK2	72.3	102.0	96.1	
Kcnj4	16520	IRK3	63.7 (67.3)	76.4 (78.8)	93.1 (70.8)	34.8
Kcns2	16539	K^+^ voltage-gated channel, subfamily S, 2	112.3	123.9	122.9	
Rims3	242662	K^+^ channel, subfamily K, member 15 and regulating synaptic membrane exocytosis 3	95.0	95.9	123.6	
Kcnt1	227632	K^+^ channel, subfamily T, member 1	83.1	95.8	80.2	
Kcnd2	16508	K^+^ voltage-gated channel, Shal-related subfamily	76.3	83.5	97.2	
Scn3b	235281	Na^+^ channel, voltage-gated, type III, beta	90.5	94.5	99.3	

The qRT-PCR data were shown in parenthesis in Table **1**. Treatment for 2 weeks with saline followed by two weeks with saline (Saline-Saline); treatment for 2 weeks with methamphetamine followed by 2 weeks with saline (MAP-Saline); treatment for 2 weeks with methamphetamine followed by 2 weeks with fluoxetine (MAP-Flx); and treatment for 2 weeks with saline followed by 2 weeks with fluoxetine (Saline-Flx). Data are presented as percentages of Saline-Saline group, representing the mean ± SEM of 4-16 determinations of pooled samples. Percentage of recovery was calculated as follows: (1-[(100-[MAP-Flx]value) / (100-[MAP-Saline]value)]) x 100.
